# Transposons-Based Clonal Diversity in Trematode Involves Parts of CR1 (LINE) in Eu- and Heterochromatin

**DOI:** 10.3390/genes12081129

**Published:** 2021-07-25

**Authors:** Anna Solovyeva, Ivan Levakin, Evgeny Zorin, Leonid Adonin, Yuri Khotimchenko, Olga Podgornaya

**Affiliations:** 1 Institute of Cytology of the Russian Academy of Science, Tikhoretsky Ave 4, 194064 Saint Petersburg, Russia; opodg@yahoo.com; 2 Zoological Institute of the Russian Academy of Sciences, Universitetskaya Nab 1, 199034 Saint Petersburg, Russia; levakin2@gmail.com; 3 All-Russia Research Institute for Agricultural Microbiology, Pushkin 8, 196608 Saint Petersburg, Russia; kjokkjok8@gmail.com; 4 Moscow Institute of Physics and Technology, Institutskiy per 9, 141701 Dolgoprudny, Russia; Leo.Adonin@gmail.com; 5 School of Biomedicine, Far Eastern Federal University, Sukhanova St 8, 690091 Vladivostok, Russia; khotimchenko.ys@dvfu.ru; 6 Department of Cytology and Histology, Saint Petersburg State University, Universitetskaya Nab 7/9, 199034 Saint Petersburg, Russia

**Keywords:** transposable elements, CR1, LINE, *Himasthla elongata*, clonal polymorphism

## Abstract

Trematode parthenitae have long been believed to form clonal populations, but clonal diversity has been discovered in this asexual stage of the lifecycle. Clonal polymorphism in the model species *Himasthla elongata* has been previously described, but the source of this phenomenon remains unknown. In this work, we traced cercarial clonal diversity using a simplified amplified fragment length polymorphism (SAFLP) method and characterised the nature of fragments in diverse electrophoretic bands. The repetitive elements were identified in both the primary sequence of the *H. elongata* genome and in the transcriptome data. Long-interspersed nuclear elements (LINEs) and long terminal repeat retrotransposons (LTRs) were found to represent an overwhelming majority of the genome and the transposon transcripts. Most sequenced fragments from SAFLP pattern contained the reverse transcriptase (RT, ORF2) domains of LINEs, and only a few sequences belonged to ORFs of LTRs and ORF1 of LINEs. A fragment corresponding to a CR1-like (LINE) spacer region was discovered and named CR1-renegade (CR1-rng). In addition to RT-containing CR1 transcripts, we found short CR1-rng transcripts in the redia transcriptome and short contigs in the mobilome. Probes against CR1-RT and CR1-rng presented strikingly different pictures in FISH mapping, despite both being fragments of CR1. In silico data and Southern blotting indicated that CR1-rng is not tandemly organised. CR1 involvement in clonal diversity is discussed.

## 1. Introduction

Transposable elements (TEs) are considered one of the main factors involved in genome reorganisation and are abundant in the genomes of many eukaryotes [1]. TE proportions vary significantly among species. TEs may comprise less than ~10% of invertebrate genomes, such as in *C. elegans*, and more than ~40% of the genomes of vertebrates, such as in humans and mice [2,3,4,5]. Based on the transposition mode and sequence organisation, TEs have been divided into two classes: class I, comprising retroelements, and class II, comprising DNA transposons [6]. Despite the development of genome sequencing and data processing technologies, TEs have been studied in only a limited number of species, predominantly higher eukaryotes. Among the existing genome annotation studies, the noncoding, repetitive parts of the genomes have received less attention than the gene-coding regions. TEs can influence the function and structure of genomes in many ways, for example, through gene sequence disorder, structural variations, altered gene expression via the regulatory elements of TEs, and epigenetic marks [7,8,9,10]. The changes that TEs introduce to the genome can lead to population isolation and, subsequently, to the formation of new species [11,12,13].

The genomes of 12 *Trematoda* species are available in publicly accessible databases. However, few trematode TEs have been recorded in the repetitive sequence database, Repbase (https://www.girinst.org/repbase/; accessed on 15 June 2019), and only a few individual TEs are described in detail.

Trematodes have complex lifecycles, and several stage-specific phenotypes are realised based on the same genome. Some stages of the lifecycle are notable for diploid parthenogenesis [14,15], which is rare among multicellular organisms (Figure S1). Clonal diversity is a known phenomenon in trematode parthenogenetic larvae. The use of PCR and Southern blot methods have led to the detection of striking interclonal and even intraclonal variations in the DNA of separate *Schistosoma mansoni* cercariae when using the tandem repetitive elements W1 [16] and W2 [17] as probes [18,19]. Other species in the Schistosomatidae and Microphallidae [20,21] families have been studied using the random amplified polymorphic DNA (RAPD) method, which revealed clonal variations. For such mutations to become fixed within different individuals, they must have occurred during the asexual replication phase while the organisms resided in snail hosts [22]. Unexpected heterogeneity was found among clonal cercariae originating from monomiracidial snail infections, so it was assumed that mitotic recombination events can occur within the heterochromatic region during sporocystogenesis [18]. The noncoding tandem repeat (TR) sequences in the W cluster may represent a template for recombination processes, but TRs are not the only or even the main driving force underlying clonal diversity. Trematode clonal diversity increases the chance of successful host penetration and further parasite transmission; thus, they seem to be a good model to evaluate the role of TEs in this event.

*H. elongata*, a marine trematode, alternates between sexual and parthenogenetic stages during its lifecycle, dwelling within different hosts: *Littorina littorea* (Gastropoda), *Mytilus edulis* (Bivalvia), and seabirds (Figure S1). When a single *H. elongata* miracidium infects a *L. littorea* host snail, it generates rediae that produce more of themselves and/or cercariae by apomictic parthenogenesis. Then, the cercariae leave the snail, locate and penetrate a second host—the blue mussel (*M. edulis*)—and transform into metacercariae. Finally, when a seabird eats infected mussels, the flukes transform into adult worms in the intestine (Figure S1). Thus, it is reasonable to expect that all parthenogenetic larvae are clones [14].

The single cercaria produced by redia are tiny with the amount of DNA measured in nanograms (body length of about 500 μm). The amplification fragment length polymorphism (AFLP) requires a low amount of DNA and the PCR amplification of genomic restriction fragments produces DNA fragments of variable size. Their quantity can be adjusted by selecting certain restriction enzyme(s). Taking into account the poor annotation of trematode genomes, the advantage of AFLP is that it is possible to obtain a repetitive and valid fingerprint set from the DNA of any origin or complexity [23]. The AFLP accuracy and reliability have been proved in a number of papers published (for example, [24,25,26]). The small size of samples and insufficient knowledge about trematode genomes makes AFLP the best tool for revealing their genetic variability.

The species *H. elongata*, which does not pose a threat to humans, has a large genome (~1 Gbp, [27]), presumably comprising many repetitive elements. Intra- and interclonal variability in infectivity rates and longevity [28], as well as in responses to light and gravity, have been observed in the larvae [29]. Clonal polymorphism in cercariae has been previously described [30], but the source of this phenomenon is unclear. In this work, we analysed the repetitive elements in the primary sequence of the *H. elongata* genome and in transcriptome data, identified clonal polymorphism in separate cercariae by using simplified amplified fragment length polymorphism (SAFLP), and annotated particular SAFLP fragments. The results provide insight into the impact of transposons on clonal polymorphism and TE organisation in trematode genomes.

## 2. Materials and Methods

### 2.1. Trematode Sample Collection

**Figure 1:**
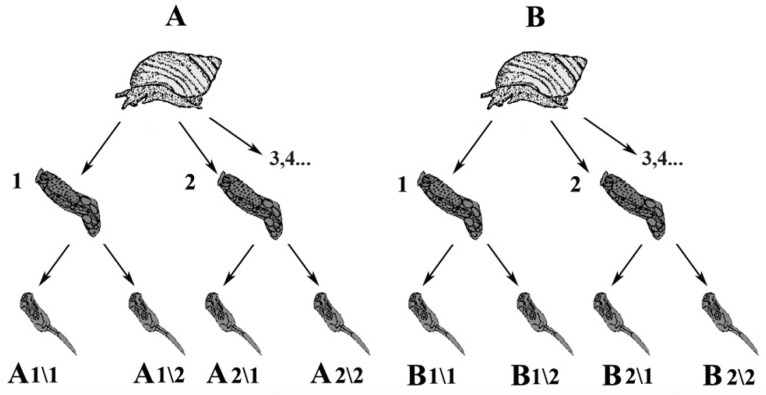
Nomenclature of clones selected for SAFLP [31]. A single miracidium infects the first intermediate host (**A**,**B**) and produces parthenogenetic generations of rediae (**A1**…; **B1**…). They reproduce themselves to maintain the population and then produce free-living larvae—cercariae (**A1**/**1**, **A1**/**2**, **A2**/**1**, **B1**/**1**, **B1**/**2**, etc.). This nomenclature is used in Figure S2.

The snail *L. littorea* is the common first intermediate host for the fluke *H. elongata* in the White Sea, where this research was carried out. Snails infected with *H. elongata* rediae were collected from the tidal zone in the vicinity of the Kartesh White Sea Biological Station of the Zoological Institute of the Russian Academy of Sciences (66°20.230′ N; 33°38.972′ E), in July–August of 2011–2014. The snails were collected from a population in which the infection rate did not exceed 1.4%. Hence, the probability of snail infection by more than one miracidium was extremely low. Cercariae were isolated from the infected snails as previously described [30,31]. Cercarial DNA was extracted with CTAB (Sigma, Burlington, MA, USA) buffer [32]. The nomenclature of the probes is shown in Figure 1. In total, three snails (A, B and C), i.e., three clonal populations (A, B and C) of *H. elongata* were studied in this work.

### 2.2. Primary Genome and Transcriptome Sequencing and Transcriptome Assembly

Total RNA was extracted from several hundreds of fluke rediae (clonal population A) with a modified AGTPC method [33] and treated with DNase I (Thermo Fisher Scientific, Waltham, Massachusetts, USA) according to the manufacturer’s instructions. The total DNA was extracted from several hundreds of redia (clonal population A) with CTAB (Sigma, Burlington, MA, USA) [32]. DNA and RNA quality control, library preparation, and sequencing were carried out at Novogene Bioinformatics Technology Company Ltd. (Beijing, China, www.novogene.cn, (accessed on 21 January 2019)) on an Illumina MiSeq (Illumina, Inc., USA) platform (2 × 150) to obtain paired-end reads. Purification of human and bacterial DNA, and rRNA in the case of RNA-Seq data, was performed with BBtools software (version 25, https://jgi.doe.gov/data-and-tools/bbtools/, (accessed on 18 February 2019)). The quality of raw reads was verified with FastQC v0.11.7 (http://www.bioinformatics.babraham.ac.uk/projects/fastqc/ (accessed on 18 February 2019)). To obtain clean reads, adaptors and unpaired reads were removed with Trimmomatic v. 0.36 [34].

The transcriptome was assembled with rnaSPAdes v. 3.11.1 [35], with default parameters. To decrease transcriptome complexity, we used the CD-HIT tool [36] to fuse identical sequences. Quality control was performed with the rnaQUAST software [37], which comprises the GeneMarkS-T [38] and BUSCO tools in its pipeline [39].

### 2.3. Repeat Content Estimation

We used the Galaxy server (https://repeatexplorer-elixir.cerit-sc.cz/galaxy/, (accessesd on 15 March 2019)) of RepeatExplorer2 to analyse the repeat content in raw genomic reads, with default parameters, and the Metazoan repeat database v3.0. The assembled transcriptome was used to generate a repeat database with the RepeatModeler software (http://repeatmasker.org (accessed on 20 March 2019)). RepeatModeler performs de novo searching of TEs based on their repetitive nature in the genome. The program creates a list of consensus repeat sequences, which can be used as a library for RepeatMasker. We conducted an additional cleaning step to eliminate numerous highly repetitive housekeeping and rRNA transcripts that were incorrectly recognised as TEs. All the sequences that coincided with proteins and did not belong to TEs were detected with the rpsblast tool and CDD (Conserved Domain Database) [40], and then removed using a custom Python script (https://github.com/NickPanyushev/IB_Himasthla/blob/master/script_filtering.py (accsessed on 25 May 2019)). We finished with 699 positively identified TEs out of the 1198 initially detected transcripts. RepeatScout, a part of the RepeatModeler pipeline, was used to perform TR prediction. Thus, we used a tandem repeat finder (TRF, [41]) to search for tandem repeats in the RepeatModeler output.

### 2.4. SAFLP (Simplified Amplified Fragment Length Polymorphism)

A simplified AFLP (SAFLP) method was performed according to Vos et al. [23], with modifications. The detailed protocol is described [31]. Cercarial genomic DNA was digested with 5 U of *HindIII* restriction endonuclease (Sybenzyme, Russia). Adapters (AdHind, AdHindR) and primers (HindIII + c, HindIII + cag) used for SAFLP are listed in Table 1.

In contrast to the standard AFLP protocol, we did not include the second restriction endonuclease *Mse*I to obtain long fragments.

### 2.5. Fingerprint Visualisation, Cloning and Sequencing

The amplified products were separated by electrophoresis in a 5% polyacrylamide sequencing gel and visualised by autoradiography [42]. Selected DNA bands (Figure S2, frames) were cut from the gel and reamplified before cloning. Successfully reamplified fragments were cloned into the pTZ57R/t vector according to the manufacturer’s recommendations (Thermo Fisher Scientific, Waltham, Massachusetts, USA). Clone sequences were produced by the Eurogene Company (Moscow, Russia).

### 2.6. Cloned Sequence Analysis

All cloned fragment sequences were analysed with the following tools: BLAST [43], RepeatMasker [44], Tandem Repeat Finder [41], and SINE Base [45]. We also compared cloned fragments with TR sets detected by RepeatExplorer2 [46] and in the RepeatModeler output. The search for transcripts corresponding to cloned fragments was carried out with the BLAST algorithm in BioEdit [47] and Usearch [48], with an E-value limit of 1.0 × 10^−40^. Kallisto [49] with default parameters was used to calculate the TPM (transcripts per million, RNA-Seq gene expression value; a normalisation method [50]). The TPM refers to the number of reads corresponding to cloned fragments in the transcriptome (Table S1). We used the NCBI Conserved Domains tool to identify any conserved motifs in the sequences (http://www.ncbi.nlm.nih.gov/Structure/cdd/wrpsb.cgi (accesed on 20 March 2019)). All cloned sequences were uploaded to GenBank (accession numbers MK287490–MK287525). To identify CR1-rng-containing sequences, we performed a BLAST search based on the whole transcriptome and mobilome contig set. All the detected sequences were annotated with Repbase, a conserved domain database, and protein BLAST.

### 2.7. Fluorescence In Situ Hybridisation (FISH)

Fluorescence in situ hybridisation (FISH) was used to map selected fragments. The probes were obtained with PCR from plasmids containing cloned fragments and labelled with biotin-11-dUTP (CR1-4_7; DNA-synthesis, Russia) and digoxigenine-11-dUTP (CR1-rng; Thermo Fisher Scientific, Waltham, MA, USA). Chromosome spreads and nuclei were isolated from rediae, and the hybridisation procedure was performed as previously described [51]. Probe signals were detected with streptavidin—Alexa Fluor 546 conjugate (Life Technologies, Carlsbad, CA, USA, dilution 1:300), and digoxigenin Alexa Fluor 488-conjugated antibody (DI-7488, Vector Laboratories, dilution 1:500) in blocking solution. The slides were counterstained with SlowFade Gold Antifade with DAPI (Molecular Probes, Waltham, MA, USA). The slides were examined with a Leica Fluorescence Microscope DMI 6000 B (Leica Wetzlar GmbH, Germany) at the Resource Centre of Saint Petersburg State University. Images were taken with a 100×/1.4 oil-immersion objective, with the appropriate filter-cubes.

### 2.8. Southern Hybridisation

**Figure 2:**
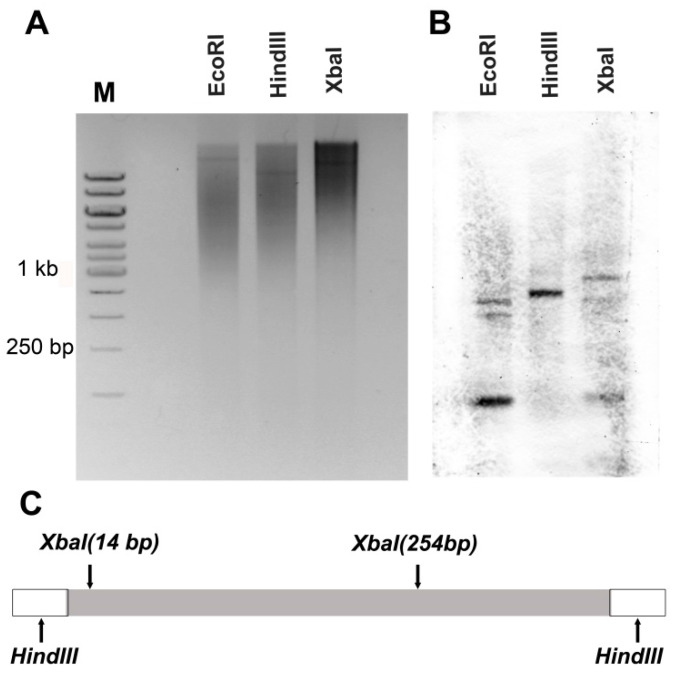
Electrophoresis in 0.8% agarose gel (**A**) and Southern blot (**B**) with CR1-rng probe on *H. elongata* DNA, digested with the indicated restriction endonucleases. (**C**) Restriction map of CR1-rng. Grey box—CR1-rng sequence, white boxes indicate flanking sequences, containing *HindIII* sites. M—DNA ladder; bp—base pairs; kb—kilobase pairs.

We used the restriction enzymes *Hind*III, *Eco*RI, and *Xba*I (SibEnzyme, Russia) to digest fluke DNA at 37 °C for 12 h. Restriction enzyme *EcoRI* does not have sites inside CR1-rng sequence, there are 2 sites for *XbaI*, and *HindIII* sites are situated at flanking sequences (Figure 2C). The restriction fragments were separated by 0.8% agarose gel electrophoresis. The DNA was transferred to Hybond N+ (Amersham, UK). CR-RT and CR1-rng fragments were labelled with biotin-11-dUTP (DNA-synthesis, Russia) by PCR, with a HindIII + cag primer (Table 2). The hybridisation occurred at 56 °C overnight, according to the standard protocol [52]. Signals were detected with streptavidin–horseradish peroxidase conjugate (Sigma, Burlington, MA, USA, dilution 1:4000) and 3,3′-diaminobenzidine, according to the manufacturer’s protocol [53].

## 3. Results

### 3.1. Primary Transcriptome and Partial Genome Sequencing

A total of 100,424,418 transcriptomic paired-end Illumina reads were generated (Bioproject PRJNA700507), of which 90,767,232 passed quality filtering and trimming, yielding 117,830 transcripts. According to rnaQUAST estimation, the assembly quality of the *H. elongata* transcriptome was comparable to published trematode transcriptomes (Figure S3). Partial genome sequencing resulted in 13,548,912 paired-end Illumina reads (Bioproject PRJNA698775), and 12,646,374 passed quality filtering. Trimmed and filtered reads were used to search for repetitive elements.

### 3.2. Repeat Content Analysis

The number of genomic reads was insufficient for draft assembly due to the lack of long mate pairs necessary for scaffold assembly. However, special tools, such as RepeatExplorer2 [46], can approximate the repetitive element content, including both tandem (TRs) and transposable elements (TEs). This computational pipeline performs raw read clustering and automated annotation and generates contigs of the identified repetitive elements or mobilome contigs. The initial transcriptome assembly was used to search for de novo TEs and TRs with RepeatModeler, which produced a database for RepeatMasker.

RepeatExplorer2 identified 191 clusters that corresponded to 44.2% of the primary sequence of the trematode genome, i.e., the number of TEs in the genome was relatively high. This proportion was of the same order as that reported for other trematode genomes (Table S2). RepeatExplorer2 did not reveal any DNA transposons, which is not typical for trematode genomes (Table S2). Based on a database generated by RepeatModeler, we performed a RepeatMasker search in the mobilome contigs and the assembled transcriptome (Table 2). DNA transposons were identified in contigs in this case. LINEs and LTRs formed an overwhelming majority of the genome and TE transcripts.

The gene-coding sequences accounted for a major part of the transcriptome, as expected; the TEs were characterised by a low level of expression compared to genes. However, the repeats constituted no less than 14.4% of the transcriptome, similar to vertebrate embryonic cells [54]. DNA transposons do not form RNA intermediates for transposition, but their transcripts are well represented in fish (lower vertebrates) transcriptomes [55]. DNA transposon transcripts are also present in trematode transcriptomes. The fraction of unclassified repeats was relatively high (~6%), presumably due to their relative species specificity [56,57]. The vast majority of transcripts represented several subfamilies of LINEs (Figure S4).

### 3.3. Description of SAFLP Cloned Fragments

TEs, detected in silico, enabled the analysis of clones obtained by cloning random fragments from SAFLP fingerprints. The obtained fingerprints showed prominent clonal diversity (Figure S2, [31]). Each lane represents a separate larva, i.e., clonal diversity was traced in individual clones. In the SAFLP protocol, DNA is cleaved with restriction enzymes, followed by adapter ligation and fragment amplification with radioactively labelled primers. We used the rare-cutting *Hind*III, which cuts within A/T-enriched sequences (gene-poor regions) and allows for determination of the cercaria fingerprint pattern presumably formed by repetitive elements. The SAFLP profiles of rediae (1) and 15 cercariae (2/1, 3/1, 4/1, etc.) were obtained from clonal populations A, B, and C (Figure S2). The most polymorphic areas are indicated by vertical lines on the right. The bulk of SAFLP fingerprints were between 200 and 1000 bp. The vast majority of prominent bands corresponded to conserved zones, with their position being the same within and between the clones. The faint bands were less conserved, and their polymorphisms were apparent within each cercaria. The cercariae possess several unique fingerprint patterns (Figure S2, black frames 4, 5, and 12). Several conserved and polymorphic bands were cut out of the gel (Figure S2, black frames 1–3, 6–11) for cloning and sequencing, resulting in 31 fragments (Table S1).

**Figure 3:**
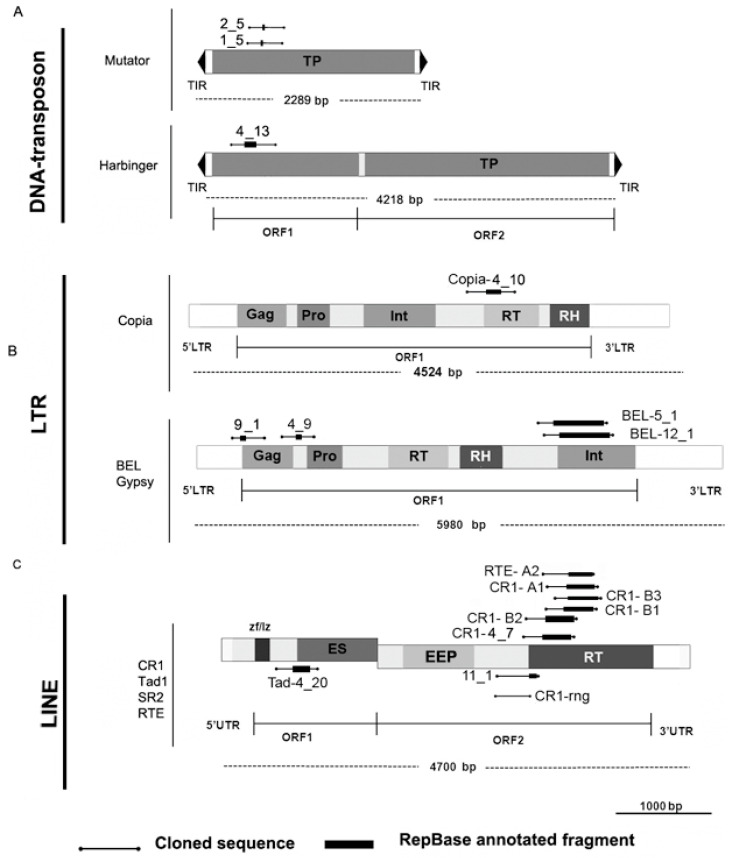
Cloned fragments aligned to TE consensus sequences. (**A**–**C**) show transposon types, to which the cloned fragments belong. Element size, 5′- and 3′-untranslated regions (UTRs), terminal inverted repeats (TIRs), long terminal repeats (LTRs), and open reading frames (ORFs) are shown. Encoded predicted proteins: zf/lz domains—“zinc fingers” and “leucine zipper”; ES—esterase, EEP—exonuclease/endonuclease/phosphatase; RT—reverse transcriptase; RH—RNAseH; Int—integrase; Gag—DNA-binding protein; Pro—proteinase; TP—transposase. TE schemes were constructed according to [58], with modifications. The similarity of each RepBase hit is reported in Table S1.

**Figure 4:**
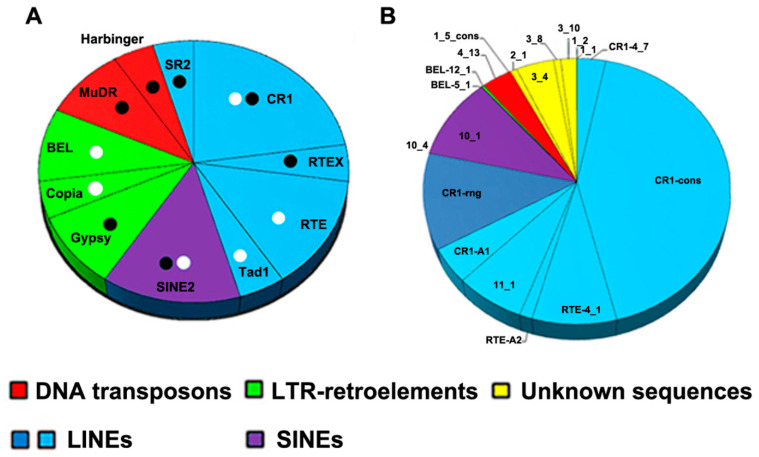
Distribution of TE families among SAFLP cloned bands from *H. elongata* (**A**), and TPM (transcripts per million) values for transcripts corresponding to cloned fragments (**B**). Similar sequences were merged into consensus sequences where possible (CR1-cons is constructed from CR1-B1, CR1-B2, and CR1-B3; 1_5_cons is composed of 1_5 and 2_5 fragments). Black dots indicate the sequences cloned from conserved SAFLP bands, and white dots indicate those from variable bands. Red—DNA TEs; green—LTR elements; light blue—LINEs (RTs containing LINE-like fragments); dark blue—LINE-like fragments containing CR1-rng; violet—SINE; yellow—non-annotated sequences.

The cloned fragments were subjected to similarity searches against the GenBank, European Nucleotide Archive, and WormBase ParaSite databases, and BLAST was performed with the assembled transcriptome and mobilome contigs of flukes. Previously sequenced fragments obtained from conserved bands after AFLP pre-amplification [31] were added to the analysis (Figure 3, non-LTR, fragments CR1-B1-B3, CR1-A1, RTE-A2, Table S1, column 1, NO. 28–31 and NO. 35). RepeatMasker was used to detect TE sequences. All sequence annotations are summarised in Table S1 and are available in GenBank (accession numbers MK287490–MK287525). Different TE coding and noncoding parts accounted for 21 out of 36 sequenced fragments. All sequences were analysed using a SINE Base search [45], and SINE-like elements were detected in several fragments (Figure 4A; Table S1). The description of the SINEs requires additional evaluation due to their high species specificity. No TRs were found among the cloned fragments with a TRF (see Materials and Methods), nor were cloned fragments found among TRs identified with RepeatModeler and RepeatExplorer2.

We compared the cloned fragments with consensus TE sequences (Figure 3). Most fragments contained reverse transcriptase (RT) domains of LINEs, and only a few sequences aligned to LTR-TE ORFs (group-specific antigen, proteinase) and LINE ORF1 (Figure 3, Table S1). DNA TEs formed a minor fraction of all sequences, and there were three sequences of Mutator and Harbinger elements in the cloned set of fragments (Figure 3, Table S1).

We did not identify a correlation between the TE type and the area of the fingerprint pattern (Figure S2), i.e., TEs of a similar family or class were found in both conserved and polymorphic zones (Figure 3A). The fragments of SINEs and LINEs were present in both conserved and polymorphic zones of SAFLP patterns. The components of LTR-TEs were found in fragments greater than 500 bp (Figure 3B; Figure S2). Most of the detected TEs belonged to the CR1 family (LINE). Two fragments had short regions of RTE and Tad1 (LINE) (Figure 3C).

Fifteen cloned fragments remained unclassified after the first step and were then used in BLAST searches of the GenBank database (https://www.ncbi.nlm.nih.gov/genbank/ (accessed on 20 May 2017)). Some unclassified fragments were present in short transcripts and the noncoding parts of trematode genomes and did not have any matches in Repbase or any ORFs. One had a high similarity score with mobilome contigs and the TE database generated by RepeatModeler from the fluke transcriptome (Table 2). We named this fragment CR1-renegade (CR1-rng, Figure 3C; Table S1, MK287521) because it corresponds to the spacer region in ORF2 of the CR1-like TE. Similar sequences were found in the genomes of the fluke species *Fasciola hepatica*, *Opisthorchis viverrini*, *Clonorchis sinensis,* and *Echinostoma caproni*.

We estimated the transcription frequency of cloned fragments using the Kallisto software, calculating the TPM values (Figure 4A,B). The non-annotated sequences are not shown on the right part of the histogram (Figure 4A), although they are robustly represented in the transcriptome (Table 2). These fragments could be species-specific TEs, since TRs are not found in the set. The highest TPM values of cloned fragments correspond to LINEs, as expected. The TPM value of CR1-rng transcription is quite high (Figure 4B, dark blue).

### 3.4. CR1-rng in Transcripts and Mobilome Contigs

**Figure 5:**
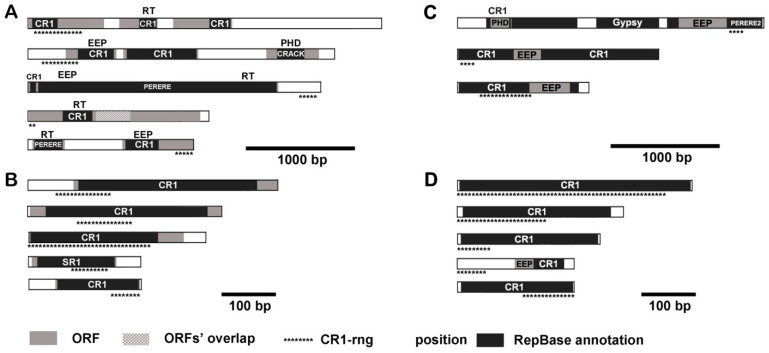
CR1-rng-containing transcripts (**A**,**B**) and mobilome contigs (**C**,**D**). Open reading frames (ORFs, grey boxes), Repbase-annotated fragments (black boxes), and transposon names are shown. Predicted conserved domains: RT—reverse transcriptase; EEP—exonuclease/endonuclease/phosphatase; PHD—plant homeodomain. Asterisks indicate the position of the match with CR1-rng. Transcripts that do not contain any conserved domains are not shown.

It was not clear whether CR1-rng transcripts were segments from the full-size CR1 or an independent element. Therefore, we conducted a BLAST search of CR1-rng related sequences in the transcriptome and mobilome contigs. The transcriptome assembly contained 35 CR1-rng transcripts. Five were larger than 1500 bp, and 11 were smaller than 500 bp. There were 19 CR1-rng-related mobilome contigs of different lengths. The longest and the shortest transcripts and mobilome contigs are summarised in Figure 5. Table S3 describes CR1-rng-related transcripts with any degree of similarity to Repbase or with conserved domains.

All CR1-rng-containing fragments possess additional parts of CR1. Most of the long transcripts and mobilome contigs had partial EEP and RT domains. These domains corresponded to Perere (Figure 5A) and CR1. None of the CR1-rng-related transcripts possessed a native ORF, i.e., their RT and EEP domains were truncated. Plant homeodomain fingers were present in one transcript and the longest mobilome contig (Figure 5A,C), and included in putative ORF1 of CR1-like elements [59]. Even fragments without positive Repbase identification showed features of EEP and RT domains (Table S3). Several sequences of the transcripts contained parts of Jockey element (Table S3).

CR1-rng transcripts appeared to bear the residues of enzymes involved in transposition, but they may exist as separate non-autonomous fragments due to the representation of short transcripts and mobilome contigs.

### 3.5. Physical Mapping of CR1-Like Fragments in the Genome

Southern hybridisation and fluorescent in situ hybridisation (FISH) revealed the genomic organisation of CR1-rng. The use of probes from RT-containing regions (RTE-A2, CR1-A1, CR1-4_7, Figure 3, LINE) on the blot resulted in an appearance typical of dispersed TEs, i.e., a smear throughout the entire electrophoretic gel, with faint zones. Dispersed LINEs appear as a characteristic smear on the Southern blot [58,60,61]. The CR1-rng probe produced distinct bands with a maximum length of ~1 kb, which indicates that most copies of CR1-rng are more than 1 kb in size (Figure 2). A characteristic ladder pattern is the result of variability or mutation within the restriction site in certain monomers which are organised in tandem. Consequently, restriction with a particular restriction endonuclease generates fragments of different length (monomers and multimers) depending on the existence or the abolishment of the restriction site within the certain monomer in an array [62,63]. On the other hand, higher-order repeat structures, which are tandem arrays of larger repeat units consisting of multiple basic repeat units, frequently give more irregular and differently structured ladders when compared to the canonical tandem repeats. However, this ladder was not observed for CR1-rng, so it is not in the TR form. The in silico data also suggest that CR1-rng is not tandemly organised: it was not found among the TRs revealed by RepeatExplorer2 or those detected in RepeatModeler output, and TRF did not reveal TRs in CR1-rng related transcripts or mobilome contigs.

**Figure 6:**
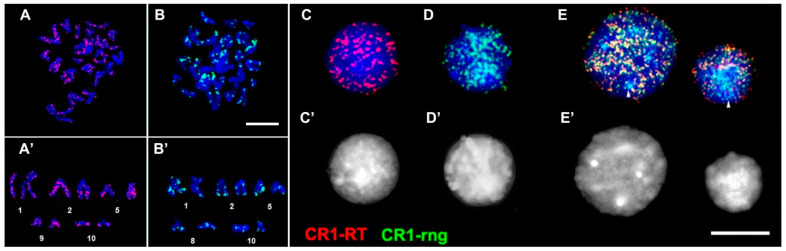
FISH mapping of CR1-4_7 (RT, red, **A**) and CR1-rng (green, **B**) probes on DAPI-stained fluke metaphase chromosomes. (**A’**,**B’**) show chromosomes with the most typical signal patterns. The same indicated probes were mapped onto DAPI-stained fluke nuclei (**C**,**D**). (**E**) Co-hybridisation of the same probes; (**C’**–**E’**)—same nuclei, shown in greyscale to make the condensed chromatin visible (bright). Scale bar—10 µm.

We mapped CR1-rng, a noncoding linker fragment, and CR1-4_7, part of the RT gene, onto fluke chromosomes and nuclei by FISH (Figure 6). Both probes revealed signal enrichment in the subtelomeric regions of chromosome 10 that bears the main rDNA clusters near centromeres [51].

Signal dispersion throughout chromosome arms is typical for LINE-like TEs (Figure 6A) and is in agreement with published data [64]. The RT probe produced the most numerous signals on chromosomes 1–5 and 9 (Figure 6A’). On the contrary, CR1-rng signals mostly localised to the subtelomeric regions of all chromosomes, with several signals near centromeres, while signals along the chromosome arms were scarce (Figure 6B,B’).

Both CR1-RT and CR1-rng fragments are part of the same CR1 element (Figure 3C), and the discrepancy in their positions was surprising. We performed co-hybridisation of CR1-rng and RT (CR1-4_7). Most signals co-localised as indicated by yellow regions in the image, which is expected for a full-length CR1 (Figure 6E). Within interphase nuclei, regions of constitutive heterochromatin were free of labels from CR-RT, but were heavily stained by the CR1-rng probe (Figure 6E). The constitutive heterochromatin regions lacked the CR1 RT probe (red), but the CR1-rng (green) signals were abundant. Some of these areas are labelled with arrows at the image periphery (Figure 6E, arrows).

Therefore, the probes CR1-4_7 (RT) and CR1-rng produced strikingly different FISH mapping results, despite both being fragments of a LINE (CR1). The CR1-rng fragment belongs to the linker region that separates two domains of ORF2 in the LINE (Figure 3C) and is in the fragment that was transcribed (Figure 4). The cloned fragments occupy different positions within the consensus (Figure 3) and reside in different parts of the genome (Figure 6). CR1-rng was initially cloned from the DNA diversity pattern of cercariae clones.

## 4. Discussion

### 4.1. LINEs in Eukaryote Genomes and Transcriptomes

Heterochromatin block variability has been detected in *H. elongata* karyotypes [51]. Heterochromatin variability could indicate the polymorphism rates in parthenogenetic clonal populations. There is a growing understanding that heterochromatin rearrangement and its tethering can be related to subtle and substantial phenotypic effects [65,66]. The identification of CR1-rng (LINE) in heterochromatin could provide insight into the mechanism of heterochromatin block variability.

The asymmetric distribution of SINEs (euchromatin) and LINEs (facultative heterochromatin) has been confirmed for most eukaryotic genomes using in silico methods [67]. In situ experiments indicate that full scale TEs of both classes are absent from heterochromatic TR-containing regions [66], but TE parts are present here. The mouse heterochromatic region (chromocenters) is enriched with a ~2 kb fragment of L1 ORF2 and 3′-UTR, named L1-htrch (L1 heterochromatin). L1-htrch is not tandemly repeated (i.e., not in the TR form) in chromocenters [68]. L1-htrch, but not the full-length LINE, is the prominent component (~11%) of mouse and human constitutive heterochromatin enriched with TR [68,69]. Murine-specific families of the LINEs LX and LX7, truncated to the same region, are also present in the chromocenter dataset. This fragment type has also been reported for the pericentromeric region in chickens: a 770 bp repeat based on a highly conserved 3′-region and a markedly truncated 5′-end of the CR1 element [70]. CR1 is considered to be an archaic element [71], and is found in many vertebrate and invertebrate genomes. The first representative of the CR1 family discovered was in the chicken genome [72]. Its 4500 bp consensus sequence comprises two putative ORFs that encode reverse transcriptase (RT), endonuclease, and a DNA-binding domain [73] (Figure 1). Most of the CR1 elements are truncated [74], and no special functions have been described for the region between RT and endonuclease domains, i.e., the linker region (CR1-rng).

Some information about CR1-rng can be drawn from the classification of LINEs in eukaryotic genomes. The 11 regions in RT are highly conserved [75], but there is a lower degree of sequence similarity within the linker region [76], which corresponds to CR1-rng in the current work. In terms of transposition, it was hypothesised that the interdomain linker regions represent a structural rather than functional component of the folded protein and, therefore, do not need to be highly conserved [76]. CR1 is not suspected to be involved in active transposition; it was used as an outgroup in the search for potentially active L1 elements. LINEs are widely represented in the genomes of known trematodes (Table S2), but no potentially active L1 elements have been found in flatworms (trematodes). Only fragments of inactive L1 copies have been identified in 11 flatworm species [71]. Thus, LINE-type TE fragmentation can be expected for the trematode *H. elongata*.

Some representatives of the LINE-type family have been mapped to chromosomes. The Rex1 group, similar to CR1, demonstrates heterochromatin-specific chromosome distribution in several lizard, frog, and bonefish species [77,78,79,80]. Two main patterns of Rex1 TE distribution have been reported: (1) enrichment in pericentromeric or subtelomeric, i.e., heterochromatic, regions [78], and (2) uniform dispersion throughout the chromosome arms [81,82,83], with some types of Rex enriched in heterochromatic regions [84]. The difference could reflect the probes’ design—whether probe developed for putative ORF or non-coding region.

Little is known about the genome allocation of trematode LINEs. Perere and Pido (CR1 family, LINE) show dispersed patterns with some low-molecular-weight bands in Southern blots, presumably corresponding to truncated copies [58,61]. FISH shows a Perere 03 signal in the subtelomeric zone in chromosome 2 of *S. mansoni* [85], similar to CR1-rng, which gravitates towards the subtelomeric regions (Figure 6).

Similar to murine L1-htrch, we found different locations for CR1 fragments, potentially a coding fragment (RT) and a linker (CR1-rng), in different regions of the trematode genome. The difference in the positions of fragments of the same TE is striking, though similar observations have been made for other LINE-type TE distributions. LINE fragments can be expected to exist in heterochromatic regions, and the current work shows that CR1-rng (LINE) is preferentially located in the heterochromatin of trematode nuclei (Figure 6). We hypothesised that CR1-rng would be integrated into tandem repeat arrays not being tandemly organised (Figure 2). This hypothesis can be tested using fibre-FISH in future work.

### 4.2. Trematode Clonal Diversity and LINE Transcription

Clonal diversity has been discovered in several trematode species using various molecular approaches [18,20,21].

The infection of intermediate host by multiple miracidia could be the probable sources of the SAFLP image looks like clonal diversity. The snails were collected in wild nature in our work. We took the precaution using rare, infected *L. littorea* population. The probability of one mollusc infection with several (n) miracidia in population can be estimated as prevalence of infection (P) to the power of n–Pn [86]. The probability of double infection counted for our case is 0.000196 for a one snail. If we consider the infection of different individuals as independent events, the probability of a double infection of three snails estimated as 7.5 × 10^−12^. This is the maximum estimate of the probability of multiple infections, i.e., this is the case of double infection of each of the three molluscs. The infection with more than two miracidia gives even less figures of probability. The negligible figures of probability make the hypothesis of multiple infection improbable. So, SAFLP image reflects the real clonal diversity.

Only one study described polymorphic fragments in the genotype patterns of trematode parthenitae. Numerous TE fragments were discovered in the RAPD profiles of *Trichobilharzia szidati* (Schistosomatidae) [87]. In contrast to our work, the authors did not find any parts of DNA TEs, but found TRs comprising ~9% of cloned fragments. Representatives of RTE and CR1 clades (LINE) were predominant both in our work and in the cited paper.

LTR and LINE fragments were non-preferentially distributed throughout both the varying and constant parts of SAFLP fingerprints (Figure 4A). CR1 and RTE-like elements (LINE) were predominant in both cloned sequences and the dataset of TE transcripts (Figure 4B; Table 2; Figure S4). Transcripts containing CR1-rng were numerous enough to form a separate section on the histogram (Figure 4B).

Most CR1-rng-related transcripts contained additional parts of CR1 (Figure 5). The existence of short transcripts may mean that CR1 is sufficient as it is. On the other hand, all long transcripts contained parts of putative coding ORFs. An example of LINE-like TE transcription exists in *Drosophila*. Jockey transcripts are generated from intraelement transcription start sites with canonical RNA polymerase II promoters [88]. This mechanism can be used for truncating transposons and adding residues of putative ORFs, as occurs in CR1-rng-containing transcripts (Figure 5).

Perere (LINE type), fragments of which exist in CR1-rng-containing transcripts, may also provide transcription machinery for truncated TEs. Perere (~4.5 kb ‘full-length’) was found in *S. mansoni* and has not been reported elsewhere, except in other trematodes. Full-length sequences were reconstructed from the transcriptome (ESTs) and have putative ORFs with several uncorrupted features, suggesting that they are possible active TEs [61]. The TE transcript frequency in cercariae was 14% of all transcripts from that stage, two-fold higher than that reported in adult flukes (Figure S1, schistosomula) and from three- to four-fold higher than that in the other stages. The active copies exhibited a 200-fold transcriptional rate per copy [61]. Cercariae are precisely the stage we used to trace clonal diversity. Perere 03 was localised in the euchromatic regions of the short arm of chromosome 2 as shown by FISH and primed in situ labelling (PRINS) [85]. Primers and probes used for Perere 03 localisation were developed to reveal active Perere 03 copies, i.e., in the RT region [89]. The heavy staining in the euchromatic regions with the RT probe in *H. elongata* (Figure 6A,A’) presumably indicates TE copies, which can produce active proteins. A high rate of Perere transcription in cercariae is observed irrespective of the copy numbers in different strains [90], which indicates its importance for this stage. The clonal diversity is produced at this stage, i.e., cercaria dwelling in rediae. It is possible that ‘full-length’ active copies of TEs provide the transcription machinery for truncated fragments, and transcripts may be involved in the heterochromatin rearrangements that produce clonal diversity. A similar scheme has been proposed for the mammalian LINE–SINE relationship [91].

In trematode parthenitae, heterochromatin is enriched with the linker fragment, and its transcripts are found in the transcriptome. Transcriptome and mobilome data suggest that different options are possible for CR1-rng appearance in heterochromatin: CR1-rng being associated to some CR1 or Perere variants (lacking RT), CR1-rng as an independent TE, CR1-rng in an independent yet truncated version, or in all the mentioned combinations. The location of TE fragments may add to genome heterochromatin rearrangement in different evolutionary groups.

## 5. Conclusions

LINEs are prevalent in mobilome contigs, the set of cloned fragments, and TEs in the redia transcriptome. Fragment corresponding to a CR1-like (LINE) spacer region (CR1-rng) was discovered among fragments involved in clonal diversity. Fragment CR1-rng could exist as an independent non-autonomous transposon dwelling in heterochromatin. According to the in silico and Southern blot data, CR1-rng is not tandemly repeated. The CR1 reverse transcriptase (RT) fragment and CR1 linker fragment (CR1-rng) are situated in different genomic regions—euchromatic (RT) or heterochromatic (CR1-rng). RT and CR1-rng are found in different transcripts, implying their involvement in mechanisms of clonal diversity in eu- or heterochromatic parts of the trematode genome.

## Figures and Tables

**Table 1:** Oligonucleotides used for SAFLP reactions, modified from Vos et al., 1995 [23].

Oligonucleotide	Sequence
AdHindF	5′-GACGATGAGTCCTGAG-3′
AdHindR	5′-AGCTCTCAGGACTCAT-3′
HindIII + c	5′-GAGTCCTGAGAGCTTC-3′
Hind + cag	5′-GAGTCCTGAGAGCTTCAG-3′

**Table 2:** Content of repetitive elements in the primary sequence of the genome and in the transcriptome of *H. elongata*.

Repetitive Element	RepeatExplorer2 Results (Predicted Proportion in Genome, %)	RepeatMasker Search in RepeatExplorer2 Assembled Contigs (% in Mobilome Assembly)	Repeat Masker Search in Transcriptome Contigs (% in Assembly)
LINE	20.85	21.75	5.27
Penelope	0.08	0.75	-
LTR	14.28	18.75	2.77
DNA transposons	-	1.95	0.63
Unclassified repeats	4.6	14.65	5.77
Simple repeats	4.39	1.5	-

## Data Availability

All scripts used in this article can be found at https://github.com/NickPanyushev/IB_Himasthla/ (accessed on 20 June 2021); raw sequencing data is submitted to Bioprojects PRJNA700507 (transcriptome reads) and PRJNA698775 (reads from partial genome sequencing).

## Supplementary Materials

The following are available online at https://www.mdpi.com/article/10.3390/genes12081129/s1, Supplementary file 1: Figure S1: A schematic lifecycle of *Himastha elongata* (Werding, 1969) and *Schistosoma sp.* (Loverde, 2019) trematodes; Figure S2: Autoradiography image of SAFLP gel carried out with redia (1) and cercariae (2/1, 3/1, etc.) from clonal populations A, B, and C; Figure S3: *H. elongata* transcriptome assembly quality evaluation; Figure S4: The distribution of transposable elements (TE) families in *H. elongata* transcriptome; Table S1: Annotation of the SAFLP cloned fragments; Table S2: Transposable elements (TE) content in sequenced trematode genomes in %; Table S3: CR1-rng containing transcripts and their composition.

## Abbreviations

TE—transposable element, LINE—long interspersed nuclear element, SINE—short interspersed nuclear element, LTR—long terminal repeat, SAFLP—simplified amplified fragment length polymorphism, AGTPC—acid guanidinium thiocyanate-phenol-chloroform, ORF—open reading frame, UTR: untranslated region, TIR—terminal inverted repeats, ES—esterase, EN—endonuclease, RT—reverse transcriptase, RH—RNAseH, Int—integrase, Gag—DNA-binding protein, Pro—proteinase, TP—transposase, TR—tandem repeat, CTAB—cetyltrimethylammonium bromide, SSC—saline-sodium citrate.
