# Supplementary material for: Transposons-Based Clonal Diversity in Trematode Involves Parts of CR1 (LINE) in Eu- and Heterochromatin
Source: Genes (Basel). 2021 Jul 25;12(8):1129. doi: 10.3390/genes12081129 (PMC8392823; doi:10.3390/genes12081129)
Supplement: Supplementary file 1 [file genes-12-01129-s001.zip › genes-1281149-SI.pdf]

Supplementary materials

Transposons' based clonal diversity in Trematode involves parts of CR1 (LINE) in eu- and heterochromatin

Anna Solovyeva[1], Ivan Levakin[2], Evgeny Zorin[3], Leonid Adonin[4], Yuri Khotimchenko[5], Olga Podgornaya[1, 6]

Author affiliations

1 — Institute of Cytology of the Russian Academy of Science, 194064 St.Petersburg Tikhoretsky ave. 4, Russia; anna_i_solovyeva@incras.ru)

2 — Zoological Institute of the Russian Academy of Sciences, 199034, St.Petersburg Universitetskaya nab. 1, Russia;levakin2@gmail.com

3 — All-Russia research institute for agricultural microbiology, 196608, Pushkin 8, Podbelsky chausse, 3, Russia; kjokkjok8@gmail.com

4 — Moscow Institute of Physics and Technology, 141701 Dolgoprudny, Institutskiy per. 9, Russia; Leo.Adonin@gmail.com

5 — Far Eastern Federal University, 690091, Vladivostok, Sukhanova st., 8., Russia; khotimchenko.ys@dvfu.ru

6 - Saint-Petersburg State University, 199034, St.Petersburg, Universitetskaya nab. 7/9, Russia;opodg@yahoo.com

* Correspondence and requests for materials should be addressed to A. Solovyeva. (email: anna_i_solovyeva@incras.ru)

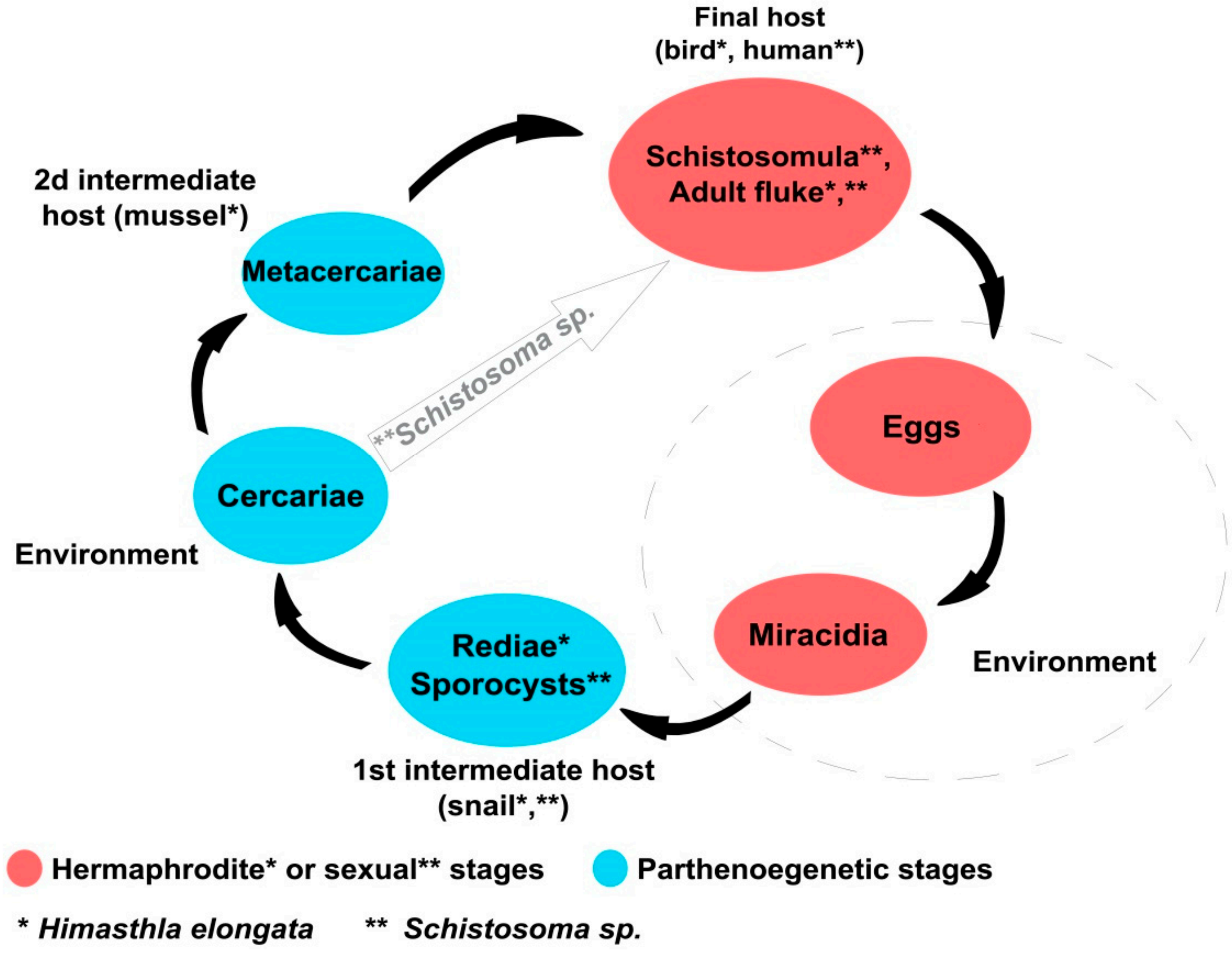

Figure S1. A schematic lifecycle of *Himastha elongata* (Werding, 1969) and *Schistosoma sp.* (Loverde, 2019) trematodes. In *Schistosoma sp.* lifecycle, cercaria transforms in schistosomula, which then becomes adult worm. Red ellipses indicate stages that have sexual reproduction or that are their offsprings. Blue ellipses indicate stages that reproduce with parthenogenesis or parthenogenetic offsprings.

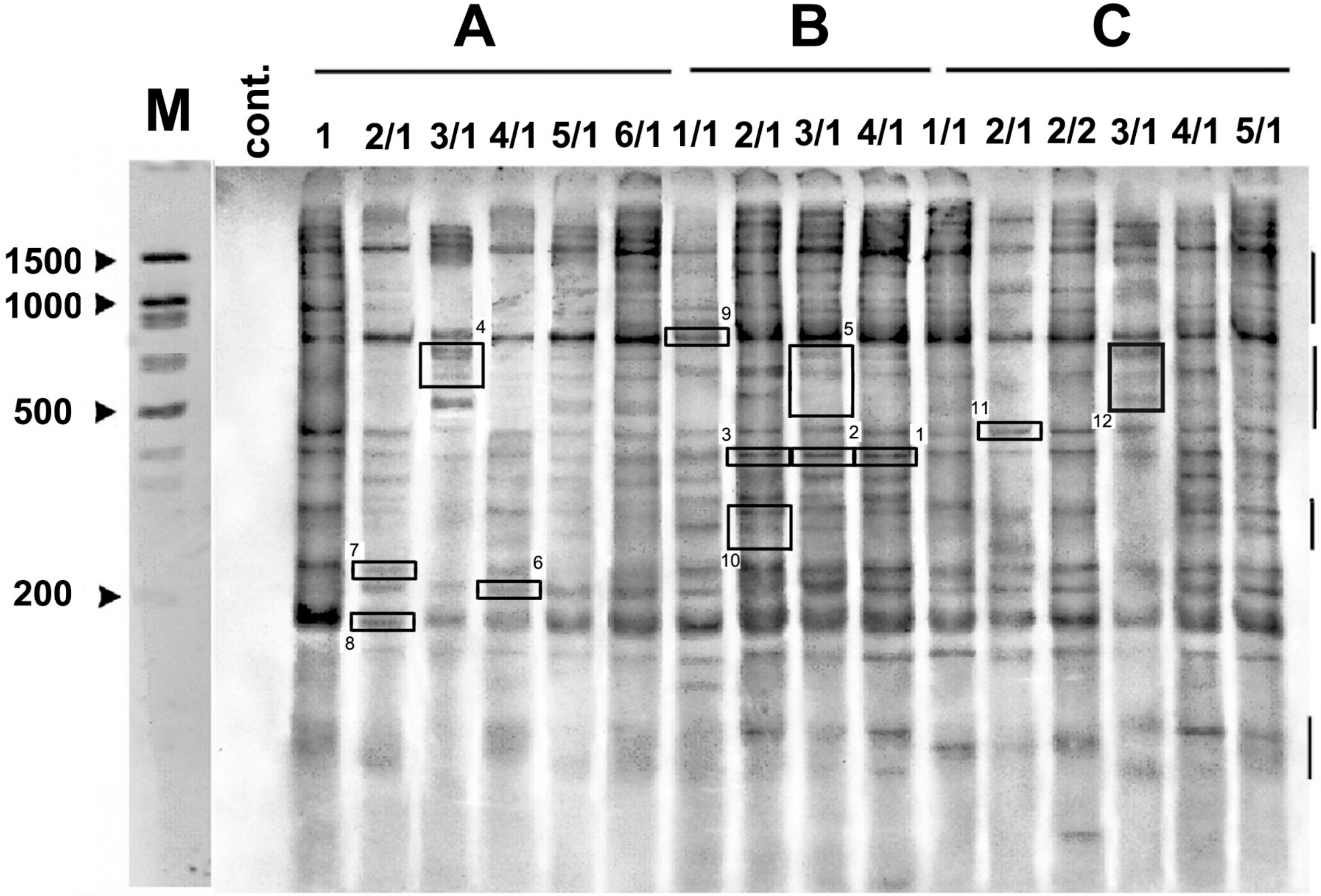

Figure S2. Autoradiography image of SAFLP gel carried out with redia (1) and cercariae (2/1, 3/1, etc.) from clonal populations A, B, and C. Vertical lines on the right indicate most variable fragments; black frames (1-12) mark the fragments chosen for cloning. M — molecular weight markers; cont. — PCR control (from Solovyeva et al., 2013 , with modifications).

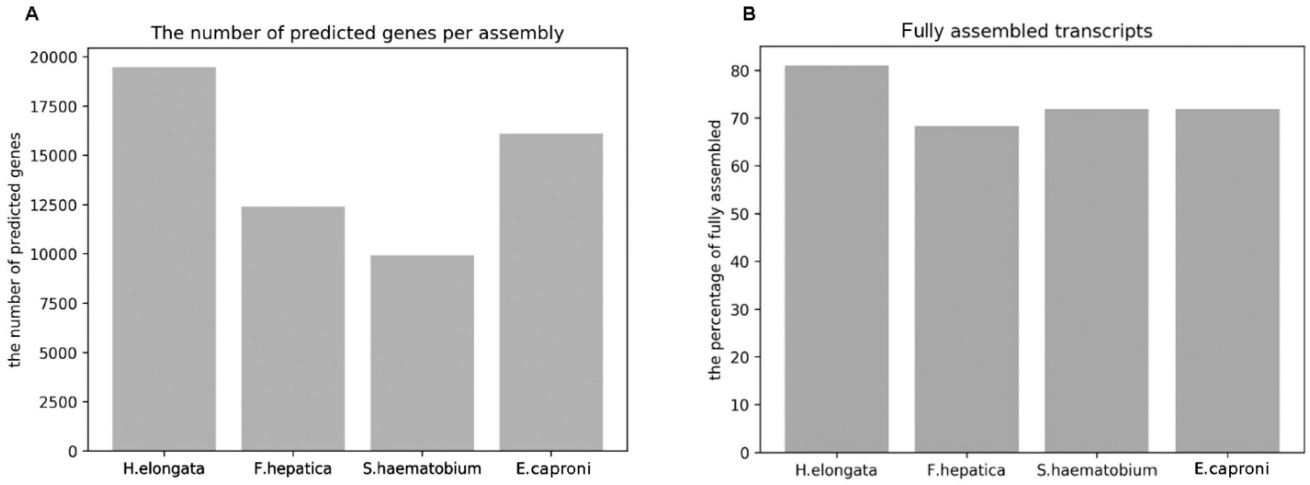

Figure S3. *H. elongata* transcriptome assembly quality evaluation. The quality of the *H.elongata* transcriptome assembly is estimated as a number of predicted genes (A) and fully assembled transcripts (B) in comparison with *Fasciola hepatica*, *Schistosoma haematobium* and *Echinostoma caproni* transcriptomes.

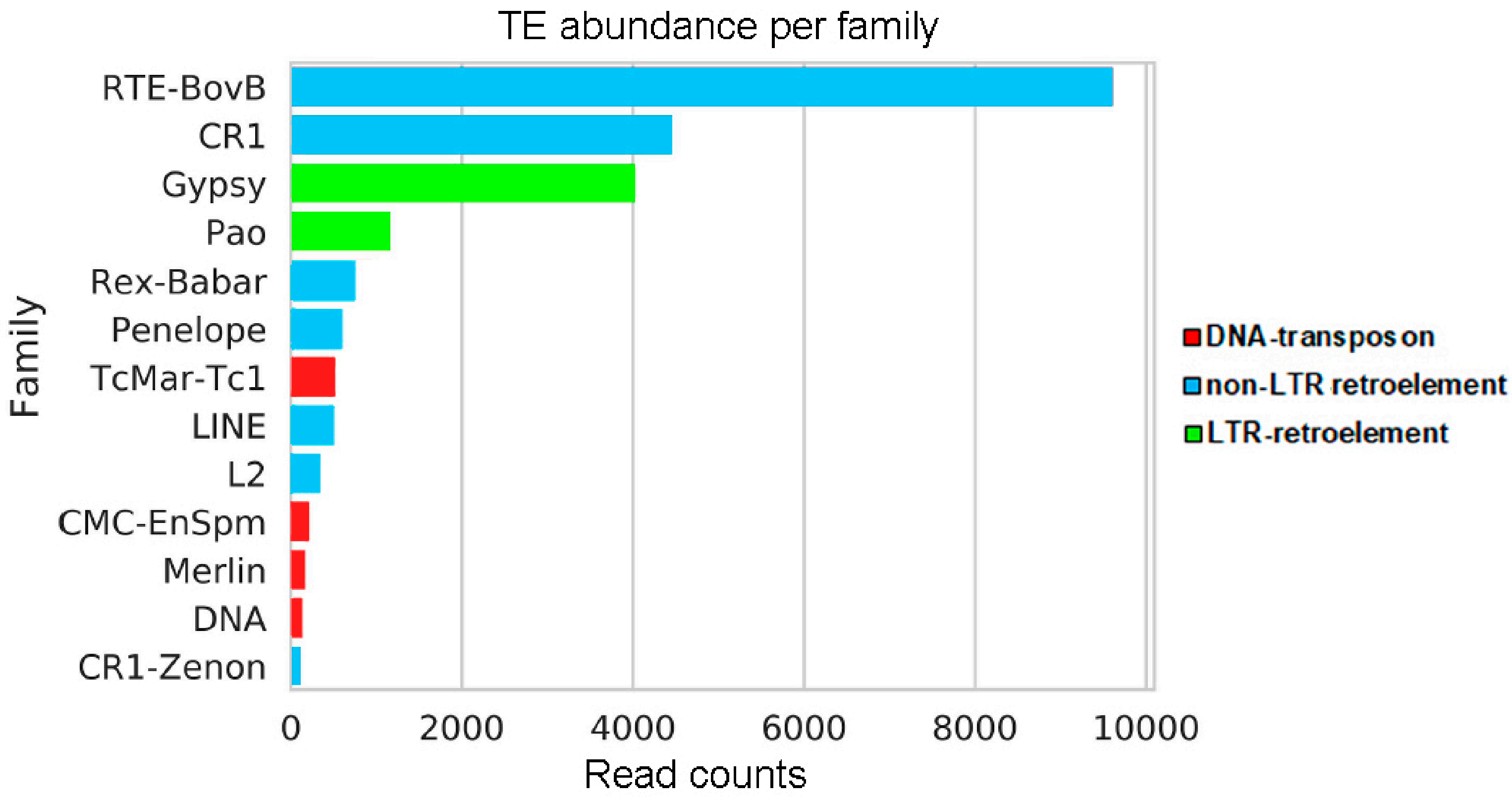

Figure S4. The distribution of transposable elements (TE) families in *H. elongata* transcriptome

Table S1. Annotation of the SAFLP cloned fragments.

№	Name	L, bp	GenBank ID	RBase	Class/family	Sim	Z	CDS	T	RGC.	TPM	Est_counts
1	1_1	563	MK287490	–	–	–	C	–	+	–	0.0008	3.26 e-05
2	1_2	530	MK287491	–	–	–	C	–	+	–	1	31.27
3	1_5	551	MK287492	MuDR-	DNA/MuDR	0.736	C	–	+	+	20.89	607.58

				1_ROr								
4	2_1	570	MK287493	–	–	–	C	–	–	+	–	–
5	2_5	547	MK287494	MuDR-1_ROr	DNA/MuDR	0.717	C	–	+	+	4837	164.11
6	3_4	296	MK287495	–	–	–	C	–	+	+	9087.06	71
7	3_5	217	MK287496	–	–	–	C	MFS	–	–	–	–
8	3_8	261	MK287497	–	–	–	C	–	+	+	1621.72	9
9	3_10	569	MK287498	–	–	–	C	–	+	+	3923.58	143
10	4_13	829	MK287499	MtPH-A6-2-Ia	DNA/Harbinger	0.710	P	–	+	+	6781.93	449.61
11	4_14	317	MK287500	–	–	–	P	–	–	–	–	–
12	Tad_4_20	498	MK287501	Tad1-35_BG	Non-LTR /Tad1	0.792	P	–	–	–	–	–
13	6_2	320	MK287502	–	–	–	C	–	+	–	6715	1
14	7_5	338	MK287503	–	–	–	C	–	–	–	–	–
15	8_1	324	MK287504	–	–	–	C	–	+	–	6715	1
16	8_3	322	MK287505	–	–	–	C	–	+	–	6715	1
17	9_1	429	MK287506	Gypsy-32_DP-LTR	LTR/Gypsy	0.766	C	–	–	–	–	–
18	9_2	413	MK287507	EptSINE1	Non-LTR /SINE2	0.750	C	–	+	+	993285	776
19	10_1	464	MK287508	EptSINE1	Non-LTR /SINE2	0.750	P	–	+	+	21725.3	530.99
20	10_4	469	MK287509	EptSINE1	Non-LTR /SINE2	0.750	P	–	+	+	0.128	0.0032
21	11_1	408	MK287510	SR2	Non-LTR/RTE	0.895	C	SLC5-6	+	+	14719.4	268
22	12_2	349	MK287511	–	–	–	P	–	–	–	–	–
23	12_3	467	MK287512	–	–	–	P	–	–	–	–	–
24	ADF-gelsolin	567	MK287513	Daphne-3_HMM	Non-LTR /Daphne	0.796	C	ADF_ gelsolin	+	–	755840	2734
25	BEL-5_1	732	MK287514	BEL-1_CSa-I	LTR/BEL	0.655	P	RVE	+	+	210.733	29.43
26	BEL-12_1	724	MK287515	BEL-1_CSa-I	LTR/BEL	0.666	P	RVE	+	+	545.148	29.57
27	Copia-4_10	476	MK287516	Copia-90_ALY-I	LTR/Copia	0.788	P	–	–	–	–	–
28	CR1_A1	542	MK287517	CR1-25_SP	Non-LTR /CR1	0.695	C	RT	+	+	4700.83	156.79

29	CR1_B1	525	MK287518	CR1-25_SP	Non-LTR /CR1	0.621	C	RT	+	+	90490.6	2841.56
30	CR1_B2	543	MK287519	CR1-X1_Pass, CR1-6_CTe	Non-LTR /CR1	0.719, 0.671	C	RT	+	+	3904.71	130.68
31	CR1_B3	523	MK287520	CR1-X1_Pass, CR1-23_HM	Non-LTR /CR1	0.724, 0.674	C	RT	+	+	28293.5	881.97
32	CR1-rng	471	MK287521	–	–	–	P	–	+	+	25041.9	632
33	CR1-4_7	525	MK287522	CR1-25_SP, CR1-X1_Pass	Non-LTR /CR1	0.617, 0.759	P	RT	+	+	6910.45	217
34	Gypsy-4_9	423	MK287523	Gypsy-32_DP-LTR	LTR/Gypsy	0.766	P	–	–	–	–	–
35	RTE_A2	475	MK287524	RTEX-14_ACar	Non-LTR /RTEX	0.750	C	RT	+	+	2724.07	70
36	RTE-4_1	931	MK287525	RTE-2_NVi	Non-LTR /RTE	0.703	P	–	+	+	18003.5	1404.39

Fragments were cloned from zones indicated by black frames 1–3, 6–11 in fig. S4; previously sequenced fragments RTE-A2, CR1-A1, CR1_B1-B3 obtained from conserved bands after AFLP pre-amplification [26, main text] added to the analysis; № — row number; name — name of cloned sequence, numbers do not correspond to the zone in the gel; L — length of the cloned fragment; Z – electrophoretic zone from which the fragment was cut: C— conservative zone, P— polymorphic zone; TPM — transcripts per million metrics, RGC — repeat contigs in genome, Est_counts — estimated counts of transcriptome reads, mapped to cloned fragments; both, TPM and Est_counts metrics, calculated with Kallisto tool. RBase — the result of search in Repbase with RepeatMasker in the overlapping sequence region between transcript and cloned fragment; Class/family – class or family for detected TE according to RepBase; Sim — similarity; CDS (Conserved Domain Search) — superfamily of conserved domains found in Conserved Domain Database in the overlapping sequence region between transcript and cloned fragment; RT — reverse transcriptase; RVE — integrase core domain.

Table S2. Transposable elements (TE) content in sequenced trematode genomes in %.

**Organism**	**Genome Size (Gb)**	**TE % in genome**	**Non-LTR elements, LINE prevail**	**LTR**	**DNA transposons**	**Other repetitive sequences, including unclassified TE**	**References**

Schistosoma mansoni	0.4	45	17	15	<1	13	[1,2]
Schistosoma japonicum	0.4	45	18	6	0.77	21	[2–4]
Schistosoma haematobium	0.37	43.26	32.65	11.24	<1%	6.94	[2,5]
Opisthorchis viverrini	0.62	30.6	13.1	5.84	3.1	11.68	[2,6,7]
Fasciola hepatica	1.14	32/55	25.45*	10.12*	4.26	16.33*	[2,8,9]
Clonorchis sinensis	0.56	25.90	10.40	1.03	2.4	14.49	[2,7,10]
H.elongata	1.2	39.81	20.93	14.28	unknown	8,99	This work

*Our data, unpublished.

References:

1. Brindley, P.J.; Copeland, C.S.; Kalinna, B.H. Schistosome Retrotransposons BT - Schistosomiasis. In; Secor, W.E., Colley, D.G., Eds.; Springer US: Boston, MA, 2005; pp. 13–26 ISBN 978-0-387-23362-8.
2. Howe, K.L.; Bolt, B.J.; Shafie, M.; Kersey, P.; Berriman, M. WormBase ParaSite − a comprehensive resource for helminth genomics. *Mol. Biochem. Parasitol.* **2017**, *215*, 2–10, doi:10.1016/j.molbiopara.2016.11.005.
3. Zhou, Y.; Zheng, H.; Chen, Y.; Zhang, L.; Wang, K.; Guo, J.; Huang, Z.; Zhang, B.; Huang, W.; Jin, K.; et al. The Schistosoma japonicum genome reveals features of host-parasite interplay. *Nature* **2009**, *460*, 345–351, doi:10.1038/nature08140.
4. Luo, F.; Yin, M.; Mo, X.; Sun, C.; Wu, Q.; Zhu, B.; Xiang, M.; Wang, J.; Wang, Y.; Li, J.; et al. An improved genome assembly of the fluke Schistosoma japonicum. *PLoS Negl. Trop. Dis.* **2019**, *13*, doi:10.1371/journal.pntd.0007612.
5. Young, N.D.; Jex, A.R.; Li, B.; Liu, S.; Yang, L.; Xiong, Z.; Li, Y.; Cantacessi, C.; Hall, R.S.; Xu, X.; et al. Whole-genome sequence of Schistosoma haematobium. *Nat. Genet.* **2012**, *44*, 221–225, doi:10.1038/ng.1065.
6. Young, N.D.; Nagarajan, N.; Lin, S.J.; Korhonen, P.K.; Jex, A.R.; Hall, R.S.; Safavi-Hemami, H.; Kaewkong, W.; Bertrand, D.; Gao, S.; et al. The Opisthorchis viverrini genome provides insights into life in the bile duct. *Nat. Commun.* **2014**, *5*, doi:10.1038/ncomms5378.
7. Ershov, N.I.; Mordvinov, V.A.; Prokhortchouk, E.B.; Pakharukova, M.Y.; Gunbin, K. V.; Ustyantsev, K.; Genaev, M.A.; Blinov, A.G.; Mazur, A.; Boulygina, E.; et al. New insights from Opisthorchis felineus genome: Update on genomics of the epidemiologically important liver flukes. *BMC Genomics* **2019**, *20*, 1–23, doi:10.1186/s12864-019-5752-8.
8. Cwiklinski, K.; Dalton, J.P.; Dufresne, P.J.; La Course, J.; Williams, D.J.; Hodgkinson, J.; Paterson, S. The Fasciola hepatica genome: gene

duplication and polymorphism reveals adaptation to the host environment and the capacity for rapid evolution. *Genome Biol.* **2015**, *16*, 71, doi:10.1186/s13059-015-0632-2.

9. McNulty, S.N.; Tort, J.F.; Rinaldi, G.; Fischer, K.; Rosa, B.A.; Smircich, P.; Fontenla, S.; Choi, Y.J.; Tyagi, R.; Hallsworth-Pepin, K.; et al. Genomes of Fasciola hepatica from the Americas Reveal Colonization with Neorickettsia Endobacteria Related to the Agents of Potomac Horse and Human Sennetsu Fevers. *PLoS Genet.* **2017**, *13*, 1–25, doi:10.1371/journal.pgen.1006537.
10. Huang, Y.; Chen, W.; Wang, X.; Liu, H.; Chen, Y.; Guo, L.; Luo, F.; Sun, J.; Mao, Q.; Liang, P.; et al. The Carcinogenic Liver Fluke, Clonorchis sinensis: New Assembly, Reannotation and Analysis of the Genome and Characterization of Tissue Transcriptomes. *PLoS One* **2013**, *8*, doi:10.1371/journal.pone.0054732.

Table S3. CR1-rng containing transcripts and their composition.

N	Transcript ID	Length	Strand (+/-)	CR1-rng position	ORFs	ORF direction	ORF position	ORF RepBase annotation	ORF conserved domains	Blast/CDD results
1	NODE_2385_length_3259_cov_15.7125_g1893_i0	3259	-	1 - 445	1	-3	701-3	CR1-3_LMi, CR1-1_LSal	-	Unkn CLF_113194 (GAA57785.1), CR1-25_BF Tektin-1(RJW61859.1)
					2	-3	1976-1389	CR1-27_BF, CR1-75_HM, PERERE-2	-	Jockey (RJW62148.1) Unkn (VDP21410.1) EEP Unkn T265_13856 (XP_009169133.1) CR1-D
					3	-2	1350-871	CR1-24_SP	Endonuclease-**RT**	Unkn CLF_104501(GAA54640.1) EEP Unkn CLF_113134(GAA57732.1) EEP Jockey (RJW71306.1)
2	NODE_3111_length_2866_cov_14.6702_g1893_i1	2866	-	470-94	1	-3	1583-996	CR1-27_BF, CR1-75_HM, PERERE-2	-	Unkn (VDP21410.1) EEP Unkn T265_13856 (XP_009169133.1) Jockey (RJW73052.1)
					2	-2	957-478	CR1-24_SP	**EEP**	Unkn CLF_104501(GAA54640.1) EEP Unkn CLF_113134(GAA57732.1) EEP Jockey (RJW71306.1)
					3	-3	2711-2274	Crack-1_NV	PHD(Plant-homeodomain) finger superfamily	-
3	NODE_3544_length_2689_cov_120.374_g1880_i1	2689	-	2566-2689	1	-2	2322-1	CR1-10_CTe PERERE-7 PERERE-4	**RT** Endonuclease/ **EEP** family	Unkn (VDP36673.1) EEP +RT TPA: endonuclease- RT (CAJ00238.1) putative RNA-directed DNA polymerase from transposon X-element (RJW68646.1)
4	NODE_7147_length_1759_cov_50.6825_g5205_i0	1759	+	40-1	1	-1	994-2	CR1-1_MUn	Endonuclease - **RT**	Unkn CLF_111752(GAA56885.1)

								PERERE-2 CR1-3b_LCh		EEP Unkn CLF_106547(GAA55043.1) EEP Jockey (RJW73052.1)
					2	-2	1731-766	DIRS-21_PSi LTR-18C_OS-LTR		Unkn DUF601 cl27239-
5	NODE_8652_length_1522_cov_47.5467_g6190_i0	1522	**+**	1341-1522	1	-2;	807-67	PERERE-2, Jockey-1_DGri	**RT**	Unkn (VDP91589.1) RT Unkn (VDP66609.1) RT TPA: endonuclease-RT (CAJ00235.1)
					2	+2	965-1522	CR1-25_BF	**EEP**	Unkn CLF_111238(GAA53824.1) EEP Unkn CLF_103784 (GAA49922.1)EEP Jockey (RJW73052.1)
6	NODE_27509_length_452_cov_359.529_g21296_i0	452	**+**	50-160	1	+2	80-451	CR1-25_SP	-	Jockey RJW61344.1
7	NODE_30251_length_408_cov_2.49292_g23978_i0	408	**+**	4-244	1	+1	34-408	-	-	Unkn CLF_112559(GAA57342.1) Unkn CLF_108868(GAA52820.1) Jockey (RJW70927.1)
8	NODE_30581_length_404_cov_1.08883_g24306_i0	404	**+**	266-357	1	+1	160-333	-	-	-
9	NODE_34554_length_359_cov_3.39803_g28263_i0	359	**+**	9-242	1	-2	328-41	-	-	-
10	NODE_35285_length_352_cov_3.81145_g28994_i0	352	**+**	210-352	1	+2	8-352	CR1-21_BF CR1-9_Crp	-	Unkn CLF_108391(GAA52583.1) Unkn CLF_104501(GAA54640.1) EEP Jockey (RJW73052.1)
11	NODE_37825_length_331_cov_3.95652_g31534_i0	331	**+**	120-221	1	+3	3-141	-	-	Unkn CLF_107365(GAA52116.1)
12	NODE_37797_length_331_cov_340.844_g31506_i0	331	**-**	230-1	1	-2	288-1	CR1-6_CTe	-	Unkn CLF_107385(GAA52133.1) Jockey (RJW73052.1) BRO1 domain-containing protein BROX(RJW63605.1)
13	NODE_65978_length_249_cov_0.979381_g59687_i0	249	**-**	242-90	1	-2	230-3	-	-	Jockey (RJW73052.1), putative serine/threonine-protein phosphatase PP2A regulatory sub-

										unit (RJW59571.1)
14	NODE_95723_length_211_cov_2.08974_g89432_i0	211	**+**	85-161	1	+3	9-167	SR 1		Unkn CLF_107249(GAA52034.1) EEP<br>Unkn CLF_101864(GAA48644.1) EEP<br>Jockey (RJW71220.1)
15	NODE_97540_length_209_cov_1.70779_g91249_i0	209	**+**	137-209	1	+1	31-207	CR1-21_BF	-	Unkn CLF_111975(GAA56996.1) EEP<br>Unkn CLF_107284(GAA52066.1) EEP<br>Jockey (RJW61935.1)
16	NODE_116140_length_150_cov_2_g109849_i0	150	**-**	150-1	1	+2	11-100	-	-	-

Table notes. Columns' names: N – number in the table; Length- fragments' lengths given in the separate column but originally included in Transcript ID in the transcriptome dataset; Strand (+/-) – CR1 used for search has orientation according to Repbase (+) and the transcript orientation given in attitude (in relation) to it; CR1-rng position – the CR1-rng position inside transcript given in transcripts' nucleotide Ns; ORFs – ORF fragments found in the transcript; Strand (+/-) – ORF orientation inside transcript; ORF position – ORF fragments position inside transcript given in transcripts' nucleotide Ns; ORF RepBase - ORF fragment annotation according to RepBase; ORF conserved domains – search shows that ORF fragment belongs to this proteins' conserved domain; Blast/CDD results – Blast search reveals the similarity of ORF fragments with the proteins indicated: **Unkn** – hypothetical protein, unnamed protein product or protein of unknown function. **Jockey** – RNA-directed DNA polymerase from mobile element Jockey; **RT** – reverse transcriptase; **EEP** – Exonuclease-Endonuclease-Phosphatase. The putative domains of the unknown proteins were determined by search in Conserved Domain Database(Marchler-Bauer et al.,2015).
